# Advances in Metabolomics-Driven Diagnostic Breeding and Crop Improvement

**DOI:** 10.3390/metabo12060511

**Published:** 2022-06-02

**Authors:** Ali Razzaq, David S. Wishart, Shabir Hussain Wani, Muhammad Khalid Hameed, Muhammad Mubin, Fozia Saleem

**Affiliations:** 1 Centre of Agricultural Biochemistry and Biotechnology (CABB), University of Agriculture Faisalabad, Faisalabad 38040, Pakistan; ali.razzaq254@gmail.com (A.R.); mmubin@uaf.edu.pk (M.M.); 2 Department of Biological Sciences, University of Alberta, Edmonton, AB T6G 2E9, Canada; dwishart@ualberta.ca; 3 Mountain Research Center for Field Crops, Khudwani, Sher-e-Kashmir University of Agricultural Sciences and Technology of Kashmir, Jammu 1900025, India; shabirhwani@skuastkashmir.ac.in; 4 School of Agriculture and Biology, Shanghai Jiao Tong University, Shanghai 200240, China; khalid_khalid45@yahoo.com

**Keywords:** metabolomics, metabolic editing, metabolomics-assisted breeding, gene-edited crops, crop improvement, climate change

## Abstract

Climate change continues to threaten global crop output by reducing annual productivity. As a result, global food security is now considered as one of the most important challenges facing humanity. To address this challenge, modern crop breeding approaches are required to create plants that can cope with increased abiotic/biotic stress. Metabolomics is rapidly gaining traction in plant breeding by predicting the metabolic marker for plant performance under a stressful environment and has emerged as a powerful tool for guiding crop improvement. The advent of more sensitive, automated, and high-throughput analytical tools combined with advanced bioinformatics and other omics techniques has laid the foundation to broadly characterize the genetic traits for crop improvement. Progress in metabolomics allows scientists to rapidly map specific metabolites to the genes that encode their metabolic pathways and offer plant scientists an excellent opportunity to fully explore and rationally harness the wealth of metabolites that plants biosynthesize. Here, we outline the current application of advanced metabolomics tools integrated with other OMICS techniques that can be used to: dissect the details of plant genotype–metabolite–phenotype interactions facilitating metabolomics-assisted plant breeding for probing the stress-responsive metabolic markers, explore the hidden metabolic networks associated with abiotic/biotic stress resistance, facilitate screening and selection of climate-smart crops at the metabolite level, and enable accurate risk-assessment and characterization of gene edited/transgenic plants to assist the regulatory process. The basic concept behind metabolic editing is to identify specific genes that govern the crucial metabolic pathways followed by the editing of one or more genes associated with those pathways. Thus, metabolomics provides a superb platform for not only rapid assessment and commercialization of future genome-edited crops, but also for accelerated metabolomics-assisted plant breeding. Furthermore, metabolomics can be a useful tool to expedite the crop research if integrated with speed breeding in future.

## 1. Introduction

In this era of global warming and unchecked population growth, food security is becoming a much more pressing concern. The dream of a world without hunger is only possible if agricultural productivity is significantly enhanced to fulfill the growing food requirements around the globe due to population growth and other factors [1,2]. It is estimated that the global population will grow from 7.8 billion today to 9.7 billion at the end of 2050. This population growth, combined with growing expectations for nutritional quality/quantity, threatens global food security [3]. Indeed, the 25% growth expected for the world’s population by 2050 will require 49% more food production by 2050. Even today, it is estimated that more than 820 million persons suffer from food insecurity and hunger. It is expected that these numbers will increase rapidly in the coming years, largely due to climate change. Indeed, many consider climate change, not population growth, to be the biggest obstacle to achieving global food security [4]. The biotic and abiotic stresses induced by global warming already adversely affect agricultural production, supply, and markets worldwide. It is estimated that global warming will lead to heavy losses of up to 50% of major cereal crops including wheat, rice, and maize by 2080 [5]. To address these challenges, crop and plant scientists must rapidly discover, design, or breed crop plants to thrive in hotter, drier conditions. This means that newer, better, and faster crop improvement tools must be found.

Metabolomics is emerging as a powerful tool for crop improvement. Metabolomics involves the comprehensive characterization of the metabolome using advanced analytical chemistry technologies. The metabolome is formally defined as the complete set of low molecular weight (MW < 1500 Da) primary and secondary metabolites found in an organism [6]. The metabolome of different organisms varies considerably according to their environmental niche and genetic complexity. Plants appear to have among the largest and most complex metabolomes. It has been estimated that the metabolome as measured across all plants in the plant kingdom contains ~600,000 different metabolites, many of which are yet to be characterized [7]. An organism’s metabolome is the product of both its genetic and environmental inputs. Therefore, measuring the metabolome of an organism allows one to explore the connections between the environment, its genes, and ultimately its phenotype.

From the perspective of plants and plant breeding, metabolomics can be readily used as a diagnostic tool to assess the plant performance, to probe vital metabolic markers linked to biotic/abiotic stresses tolerance, perform mutant characterization, and conduct robust ecotype detection [8]. Metabolomics has largely been focused on the identification of metabolites using standard analytical chemistry platforms such as nuclear magnetic resonance spectroscopy (NMR), liquid chromatography mass-spectroscopy (LC-MS) and gas chromatography-mass spectrometry (GC-MS) [9]. In most cases, traditional metabolomic analysis has been relatively manual, less sensitive, and labor intensive with very small metabolome coverage and less accurate in the identification of metabolites in any given study [10]. It is also quite insular in that metabolomics rarely links metabolomic data to other “omics” data due to outdated analytical software [11]. The advent of more sensitive, more automated and higher throughput analytical tools (for metabolomics) combined with advanced next-generation DNA sequencing, sophisticated bioinformatics tools, and high-throughput phenotype screening techniques has led to the advent of advanced metabolomics. Now, metabolomics studies can offer maximum spectrum coverage to any plant metabolome and detect/identify a large number of metabolites using multi-platform analytical tools and also those studies that combine advanced metabolomics tools with other omics technologies, including genomics, transcriptomics, proteomics, and other molecular phenotyping techniques to guide the metabolomic research [12,13]. In conjunction, other “omics” technologies can provide the more holistic, multi-dimensional, multi-omics datasets that are crucial to dissect and understand the relationship between genotype metabotype and phenotype [14,15]. Furthermore, it allows scientists to rapidly map specific metabolites to the genes that encode their metabolic pathways and can provide a roadmap to follow a specific biological process starting from gene level to its end product, metabolic level. It also offers plant scientists the opportunity to more fully explore and harness the wealth of metabolites that plants biosynthesize and the genes that encode them [16]. The more sophistication in metabolomics will allow crop scientists to pinpoint crucial metabolic pathways and to acquire a comprehensive understanding of these pathways. This may lead to the metabolic editing or engineering by targeting multiple genes governing a specific metabolic response simultaneously.

**Figure 1:**
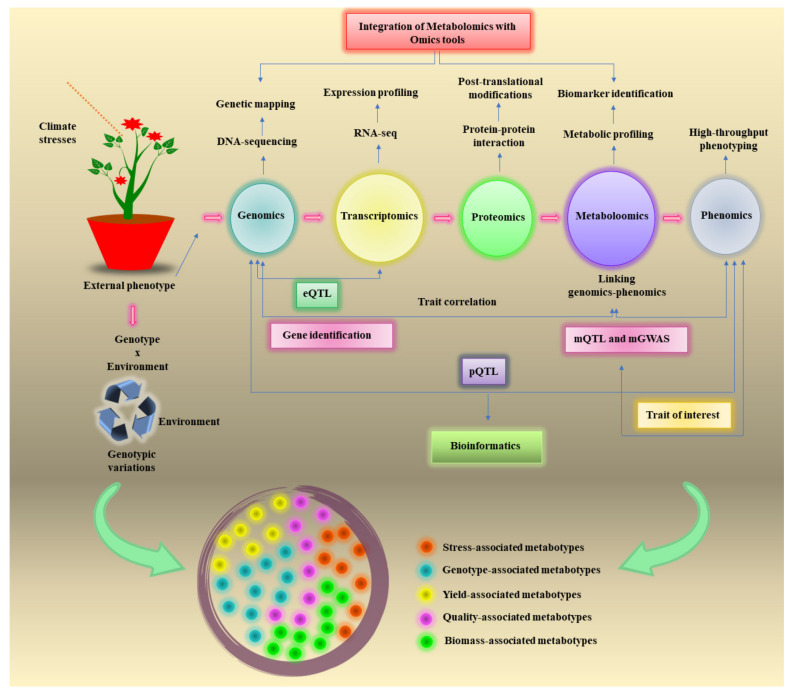
An illustration of the applications of metabolomics for crop improvement. Metabolomics can be integrated with other omics tools to elucidate the molecular phenotype corresponding to the desired trait of interest and to assist in the mapping of unique genes regulating different metabolic pathways under different conditions (we use climate stress as an example). Molecular information collected from genomics and phenomics can be used to correlate traits or genes through quantitative trait loci (QTL) and genome-wide association studies (GWAS). Metabolomics-based QTL (mQTL) and metabolomics-based GWAS (mGWAS) measure variations in molecular phenotype without requiring any genetic information, and thereby remove the genotype–phenotype gap efficiently.

In this review, we will discuss the applications of advanced metabolomics for crop improvement (illustrated in Figure 1). We elaborate on metabolomics-assisted breeding and show how it is being used in conjunction with other omics tools to decipher abiotic and biotic stress tolerance mechanisms in major crop species. In addition, we highlight some of the emerging trends in metabolic editing using metabolically guided multiplex genome editing technologies. In particular, we will describe a number of recent studies aimed at plant metabolic editing via CRISPR/Cas9 technology and how untargeted metabolomics could be used as a vehicle for not only guiding the editing process by performing the necessary risk assessment of these metabolically edited crops. These and other examples should help illustrate the progress that is being made to fully exploit metabolomics in optimizing the metabolic potential of crops and in facilitating rapid regulatory approval of genome engineered crops. Finally, we describe how the integration of “speed breeding” with metabolomics could greatly expedite crop improvement programs in future.

## 2. Linking Genomics to Phenomics through Metabolomics-Assisted Breeding

Metabolomics provides an excellent platform for diagnostic breeding to screen and select elite crop cultivars with improved stress tolerance [17]. It also offers an opportunity to discover hidden regulatory networks that control crop development and other important agronomic traits at a metabolic level [18,19]. A comprehensive understanding of how gene networks control key crop traits such as crop quality, yield, and stress resilience would allow plant breeders to select and produce what are now called “climate-smart” crops. Today breeders are facing unprecedent new challenges particularly, identification of novel traits that can guarantee greater stress resilience and higher crop yields in response to extreme biotic/abiotic threats [13]. However, the main bottleneck to selecting optimal crop traits for these climate-smart crops is the limited availability of genotype–phenotype information [7]. Understanding the genetic basis of these complex traits and introgression of desired traits into new lines required integrated strategies for sustainable agriculture production.

**Figure 2:**
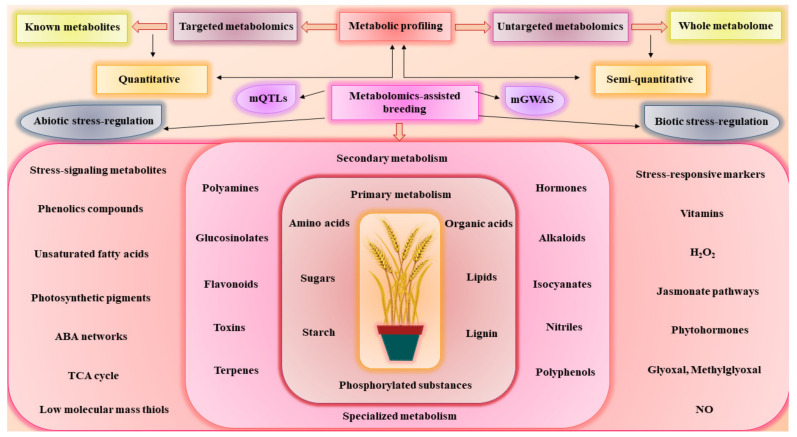
Overview of metabolomics-assisted breeding using metabolomics tools to study abiotic and biotic stress regulation in plants. Primary metabolism produces essential metabolites which are necessary for the plant growth and development, while the secondary metabolism produces specialized metabolites that are triggered by exposure to various stressors. These stress-induced metabolites are crucial for plants to adapt to harsh environmental conditions.

Metabolomics is particularly well-suited for acquiring genotype–phenotype information and detecting the metabolic diversity within a species [20]. Integration of metabolomics with other omics techniques has gained popularity to understand the system biology and can elucidate the genotype–phenotype linkage [21]. It has provided exceptional opportunities to take crop breeding into new heights by allowing to expand the genetic pool, identification of novel genes, introgression breeding countless agronomic traits, detailed phenotyping, stress tolerance, and its corresponding metabolic trait discovery [16]. The retrieval of huge datasets from integrated platforms offers a key platform to establish a statistical modeling system to study the genotype–phenotype relationship through genome-scale-based metabolic reconstruction [22,23]. Furthermore, the functional association of genes in the secondary metabolism needs to be studied by capturing the complex metabolic pathways. For example, Feng et al. [24] conducted the integrated metabolic and transcriptomic analysis to study the metabolites concentration and gene expression for organic acids and sugars in pomegranate. The results demonstrated that some genes such as *HK1, PFK7, FRK7* and *INV2* were found to regulate the glucose and fructose contents. In recent years, this integration achieves more sophistication in functional genomics to annotate the genes functions [13]. Therefore, metabolomics can be readily applied to detect the metabolic consequences of altering or adding novel plant genes. This helps bridge the gap between genotype and phenotype, as depicted in Figure 2.

Historically, quantitative trait loci (QTL) studies and genome wide association studies (GWAS) have been the traditional approaches to identify broad, macroscale phenotype-to-genotype relationships. However, the macroscale phenotypes used in these studies (such as yield, growth rate, disease tolerance, and taste) are often “ambiguous” or not clearly defined, as they represent the downstream effects of hundreds of genes, proteins, and metabolites. These unclear effects are compounded by difficult-to-measure environmental and epigenetic effects. As a result, the apparent strength or influence of these genetic effects on macroscale plant phenotypes is greatly diminished or difficult to detect. By focusing on molecular-scale phenotypes, such as metabolite concentrations that can be quantitatively measured, one can identify more compelling and higher performing gene-phenotype relationships. As a result, metabolic qualitative trait loci (mQTLs) studies and metabolic genome-wide association studies (mGWAS) have emerged as powerful tools (Figure 1) to dissect the relationship between genotype and phenotype and to decipher genetic variants associated with metabolic traits (Table 1) [25]. Different environmental stimuli result in the genetic diversity and genomic evolution which led to an increase in the metabolic diversity. These approaches give in-depth information about the genetic variation in phenotypes due to the changing genetic architecture by correlating the gene expression with metabolite profiles.

For example, mGWAS analysis has proved to be crucial for dissecting the genetic and metabolic architecture of rice by identifying the genes associated with the natural variation seen in the rice metabolism. GWAS was used to identify 6.4 million SNPs from 529 different rice strains and identified 36 candidate genes that modulate metabolite levels of at least 34 known primary and secondary metabolites. The results showed that this technique can be crucial to perform molecular phenotypic trait mapping for the goal of rice improvement [26]. Yadav et al. [27] performed metabolomic fingerprinting of 197 pearl millet inbred lines using flow infusion high-resolution mass spectrometry (FIE-HRMS) and detected various metabolite features associated to nutritional benefits like lipid metabolism, vitamins, antioxidants, and dietary starch. mGWAS analysis identified 897 SNPs and 738 candidate genes having function in the nutritional value of pearl millet.

A comparative analysis using a combination of phenotypic genome-wide association studies (pGWAS) and mGWAS was carried out on a diverse collection of rice accessions. LC-MS/MS-based metabolomic analysis allowed the identification of more than 830 metabolic features having high heritabilities for flavonoids and amino acids. These results indicated a relationship among phenotype-metabolites that can be beneficial for complex phenotype trait mapping [28]. Matros and co-workers [29] conducted a metabolomics study on 135 different winter wheat lines via targeted GC-MS techniques and identified 75 metabolites. This metabolomic analysis was combined with a 90k SNP array mGWAS analysis that measured 17,372 SNPs. It was noted that the abundance of oxalic acid plays a role in aluminum tolerance/detoxification for wheat. This data provides further support that marker-assisted breeding through metabolites is becoming feasible to crop improvement. mGWAS can offer global detection of these unique genetic determinants controlling the metabolic pathways and provide detailed knowledge about their genetic bases. Still, variation in single metabolite content within the cell and its genetic bases cannot be fully understood by using mGWAS analysis, even though several vital genes and gene networks controlling the metabolic diversity have been identified using mGWAS multidata analysis [25].

## 3. Metabolomics-Assisted Breeding for Agronomics Traits

Metabolomics-based mGWAS analysis for individual metabolite content rations subjected to climatic stresses will give deep insight and knowledge about metabolic diversity and discover key metabolic regulators in response to different stresses. For instance, LC-MS/MS-based metabolomics was used to perform untargeted metabolic profiling of 282 inbred lines (ILs) of maize. This led to the identification of 3991 mass features that were then compared to >29 million SNP markers. Numerous hotspots for QTLs were identified that control a citrate synthase-like gene and benzoxazinoid biosynthesis genes that may play key roles in the synthesis of pathogen defensive metabolites. The genetic loci identified would be useful for marker-assisted breeding to develop resistant maize lines [30]. LC-MS based metabolomics was used for profiling 266 maize inbred lines under salinity stress and detected various salt tolerance metabolic markers. mGWAS analysis revealed 10 corresponding genes that provide salt tolerance and can be useful in genetic improvement of maize [31].

Liu and coworkers analyzed 286 soybean varieties with a SNP chip that identified 54,294 SNPs. This SNP analysis was combined with a targeted metabolomic study that identified 52 metabolites for lipid metabolism and six oil-related traits. Additionally, multi-omics analysis identified 284 genes associated with oil-related traits. This work also allowed the authors to construct 133 genetic networks related to soybean metabolism, of which only 24 were previously known [32]. Target metabolic analysis of 150 millet accession was carried out using an LC-ESI-MS/MS platform and identified 330 annotated metabolites with significant variations in lipids, hydroxycinnamoyl derivatives, phenolamides, and flavonoids. mGWAS was used to analyze the genetic basis of these metabolites and identified two candidate genes: quercetin O-acetylhexside and cyanidin 3-O-glucoside [33]. Chen et al. [34] performed mGWAS on wheat kernels to identify a major flavonoid synthesis pathway. Using LC-MS/MS-based metabolomics, the Chen team identified 805 metabolites from the mature seeds of 182 wheat genotypes. Using a collection of nearly 15,000 SNP markers, they were able to generate 1098 highly significant mGWAS associations. This led to the identification of 26 candidate genes for 42 different loci that can assist the future programs of wheat breeding. Zhu et al. [35] conducted a multi-omics experiment on 610 tomato accessions and found 13,000 triple-associations between genes + SNPs + metabolites in their mGWAS and eQTL analysis. Resequencing analysis identified 26,273,368 SNPs and 33,088 genes. Metabolomics analysis was performed using targeted LC-MS/MS and identified 980 putative metabolites in the pericarp of ripened tomatoes. This multi-omics study revealed some of the key determinants responsible for metabolome diversification in tomatoes. It also provided an improved understanding of the possible effects of domestication and breeding on the metabolomic features of tomatoes.

Advances in genomics and metabolomics technologies have allowed mQTL mapping for diagnostic markers identification to study the plant performance and enabled to develop high density maps of candidate genes controlling the various metabolic pathways [43]. Metabolic markers can serve as promising diagnostic tool to unveil the hidden biological processes controlling the specific phenotypes under different environments. The mQTL technique integrates the metabolomics with other omics approaches that uncover distinct genetic functions and examine the plant phenology via gene expression analysis and metabolic profiling [36]. Integrated QTL mapping and metabolic profiling provide an excellent approach to study the genetic diversity of secondary metabolites under stress conditions. LC-MS-based profiling of barely inbred lines demonstrated the production of several stress responsive metabolites under drought stress [37]. Templer and colleagues [38] studied the environmental and genetic control of metabolic adaptation of diverse group of barley lines subjected to drought and heat stress and found many stress-related mQTLs for succinate, glutathione, and γ-tocopherol in flag leaf and can be useful for breeding elite barley genotypes. Alseekh et al. [39] studied the canalization tomato using introgression lines and detected numerous mQTLs that cause variations in primary and secondary metabolites due to genotype × environment interaction. In an earlier experiment, Alseekh and co-workers [40] used introgression and wild lines of tomato and identified 679 mQTLs regulating the complex secondary metabolism pathways which provide environmental stress tolerance.

Shi et al. [36] examined the wheat kernel metabolome via LC-MS/MS and mQTL techniques to dissect the genetic relationship between metabolites and agronomic traits. About 1260 metabolic features and 1005 localized high resolution mQTLs were identified. A total of 24 candidate genes were found to be involved in the synthesis of flavonoids and control number of grains per spike and plant height. Selecting wheat plants that show increased expression of these metabolites could contribute to the improvement of wheat yields. The nutritional quality of the strawberry was studied by dissecting the flavonoid-related traits via LC-ESI-MS which identified different compounds like phenolics, flavonoids, and anthocyanins. A total of 178 mQTLs were detected, associated with flavonoids-related pathways that can be used for marker-assisted selection of strawberry for improved nutritional quality [41]. Metabolic diversity at various growth stages have been captured by performing the metabolic profiling of rice seeds. A total of 210 recombinant inbred lines were used to make high density genetic maps and discovered 4681 mQTLs and 35 candidate genes responsible for the metabolic variations and agronomic traits in rice [42].

## 4. Integration of Metabolomics with OMICS Tools for Climate Resilience

The mGWAS and mQTL studies outlined earlier are now giving plant scientists a much clearer idea about the metabolic genes and metabolic gene networks involved in a variety of metabolic activities for many important crop plants. This set of improved functional genome annotations is opening the door to performing much more focused metabolic profiling. In particular, metabolomics is now being extensively used to study plant responses to biotic and abiotic stress. This is helping to elucidate the unique metabolic pathways and metabolic biomarkers linked to stress tolerance and disease resistance in globally important crop plants. In many cases, the pathways and genes associated with these metabolic biomarkers to stress are now well known.

Suharti et al. [44] used two different rice genotypes (32R and 29S) infected with the common plant pathogenic fungus *Rhizoctonia solani*. This metabolomic analysis revealed an increased production of ADP, mucic acid, jasmonic acid, and glyceric acid in resistant cultivars (32R), while sensitive cultivars (29S) increased their production of inosine monophosphate. These data suggest that different metabolic strategies are adopted by different rice cultivars to fight *R. solani* infection and that metabolite-based selection and breeding for increased levels of these pathogen-fighting metabolites may improve rice resistance to this widespread fungus. The ascomycete fungal pathogen known as *Fusarium graminearum*, is a common crop pathogen that causes fusarium head blight (FBH). FBH is a devastating disease specific to wheat and barley and is responsible for billions of dollars in economic losses worldwide each year. With climate change and global warming, FBH in wheat is of increasing concern. FBH infection causes shifts in the amino acid composition of wheat, resulting in shriveled kernels and contaminating the remaining grain with mycotoxins, mainly deoxynivalenol, which inhibits protein biosynthesis, and zearalenone, an estrogenic mycotoxin. These toxins cause vomiting, liver damage, and reproductive defects in livestock. They are also harmful to humans when they appear in contaminated food [45].

In a study described by Dhokane et al. [46], recombinant inbred lines (RILs) of wheat were subjected to integrated metabolomics and transcriptomics to identify potential FHB resistant genes in the *QTL-Fhb2* allele. Untargeted metabolomics showed increased accumulations of fatty acids, flavonoids, glycerophospholipids, lignins, phenylpropanoids, and terpenoids in FHB resistant RILs. In addition, transcriptome profiling detected numerous mycotoxin detoxification genes, transcription factors, receptor kinases, and disease resistance genes. By combining these datasets, several candidate resistance genes within the *QTL-Fhb2* allele, including cinnamyl alcohol dehydrogenase (CAD), 4-coumarate—CoA ligase (4CL) and ABC transporter-4 (ABC4)—were identified.

In a related work, Cuperlovic-Culf et al. [45] conducted NMR-based metabolic profiling on the infected spikelets and rachis of wheat cultivars. Their results showed that higher concentrations of γ-aminobutyric acid (GABA), spermine, lactic acid, and putrescine could be found in resistant plants when infected with *Fusarium graminearum*. These metabolites can potentially be used as disease resistant biomarkers, or they may be used in metabolite-based breeding studies to select for increased levels of these pathogen-fighting metabolites. Indeed, the genes and metabolic pathways in wheat are already known for GABA, spermine, lactic acid, and putrescine production [47].

While many resistance genes against wheat FHB have been identified, we still lack a good understanding about the stress-regulation mechanisms associated with these genes. Recently, Su and colleagues [48] employed integrated metabolomics and transcriptome profiling to study FHB infected wheat lines and identified 789 metabolites producing in varying concentrations including phytohormones, tryptamine derivatives, phenolamides, and flavonoids. The transcriptomic data also revealed the differential expression of about 100 genes that control the biosynthetic machinery of FHB-resistant pathways. Additionally, the effect of FHB infection on auxin and flavonoid concentrations was studied by mutating or silencing (via RNAi technology) the *TaTIR1* gene. This result and the multi-omics data produced by this study provide a much more detailed mechanistic understanding of the wheat response to *F. graminearum*. Kim et al. [49] performed transcriptomic and metabolomic analysis to understand the regulating response of *Rp1-D* gene against *P. sorghi* The results showed that these defensive genes involved in secondary metabolism including terpenoid, flavonoids and phenylpropanoid pathways. Furthermore, many transcriptional factors such as MYB100, BZIP84, and WRKY53 were detected as targeted metabolites for specific stress response.

Karre et al. [50] performed integrated analysis using genomics, transcriptomics, and metabolomics. They found higher concentrations (relative to disease-susceptible strains) of a number of metabolites including jasmonic acid, hydroxycinnamic acid, and several phenylpropanoids. They also identified changes in their corresponding genes including MAP kinase 3 (*HvMPK3*) and chitin elicitor receptor kinase (*HvCERK1*). Furthermore, knockouts of *HvCERK1* gene in the tolerant barley genotype showed how important its role was in the FHB resistance mechanism and in the biosynthesis of disease-resistant metabolites.

Kernal bunt (KB) is another economically important disease of wheat caused by smut fungus (*Tilletia indica*). KB is a floret-infecting disease that damages wheat kernels and causes them to emit a pungent fishy odor arising from the fungal teliospores. This obviously reduces grain quality. In a multi-omics study, MALDI-TOF/TOF mass spectrometry was employed to perform a proteomic analysis of highly virulent and weakly virulent isolates of *T. indica*. This led to the detection of 21 proteins that were differentially expressed in the highly virulent isolates. Protein and DNA sequencing data from the identified *T. indica* proteins led to the determination that certain candidate pathogenicity proteins were expected to produce oxalic acid. This illustrates how integrated tools that combine proteomics with metabolomics offer an excellent platform to detect virulency-associated compounds in plant pathogens [51].

A number of metabolomics studies have also been undertaken to look at pathogens and biotic stressors in other crops, including beans and potatoes. Chen et al. [52] used de-novo transcriptome and metabolic profiling to analyze the root metabolome of common beans infected by *Fusarium oxysproum f. sp. Phaseoli*. RNAseq data showed the differential expression in transcript levels for a number of pathogenesis-related genes between infected a non-infected plants while UPLC-MS based metabolic analysis demonstrated a significant variation in metabolites associated with the ethylene, jasmonate, salicylic acid, and flavonoid synthesis pathways in response to the *Fusarium oxysproum* infection.

*Phytophthora infestans* is the causative agent of potato blight, which is widely considered to be the most devastating potato disease around the globe. The disease resistance mechanisms are poorly understood, as there are numerous molecular and biochemical pathways involved in the process. To address this issue, Yogendra and Kushalappa [53] applied both transcriptomics and metabolomics to elucidate molecular and regulatory changes taking place after *P. infestans* infection in tolerant and susceptible potato varieties. Transcriptomic studies detected 4216 genes that showed differential expression levels in resistant lines as compared to vulnerable lines. Untargeted metabolomics using LC-HRMS detected 4811 metabolites or metabolite features, of which 589 were identified. The most significantly changed metabolites included terpenoids, alkaloids, flavonoids, and phenylpropanoids, many of which were associated with the genes identified in the transcriptome arm of the study. This work has given important new insights into the disease resistance mechanisms associated with potato blight and is suggesting new approaches to select for disease-resistant strains.

In addition to looking at metabolite changes due to biotic stress in crops, the effects of abiotic stress have also been studied through metabolomics [54]. Metabolomics provide a significant achievement in studying the wheat metabolome to give breeders a much clearer understanding that assists them to develop elite wheat cultivars [55]. For example, a GC/MS-based metabolomic study published by Kang, et al. [20] detected and quantified 142 metabolites in the flag leaves and 99 in the roots in the two different varietals. After this drought stress, much greater metabolite variations occurred in the leaves as compared to the roots of the drought-tolerant variety. In particular, the concentrations of malic acid, fumaric acid, citric acid, valine, and tryptophan were found to be increased in the leaves while they were downregulated in roots. Untargeted metabolomic assays on wheat leaves detected 691 metabolites, of which 175 were identified with high confidence. The results showed a two-fold higher production of several phenol-containing compounds including picolinic acid, pyridoxal, alpha-phocaecholic acid, vanillin, and homovanillic acid in the tolerant variety [56].

The hyper-production of primary metabolites including specific amino acids such as branched-chain amino acids (BCAAs) also appear to provide a defense mechanism against severe environmental stresses. For example, in a study, integrated metabolomics and transcriptomics were used to study the BCAT genes and their corresponding metabolites in two durum wheat genotypes. Molecular characterization and expression analysis of the BCAT genes revealed the crucial role of the *TdBCAT* gene in drought tolerance at the flowering and grain filling stage, having increased levels of BCAAs (valine, leucine, and isoleucine) in the tolerant genotype. These findings highlight the benefits of integrating metabolomics and transcriptomics in next-generation breeding programs to create climate-resilient varieties [57].

The malting quality of barley can be degraded by post-anthesis drought stress. This can significantly enhance the β-amylase and grain protein and decrease β-glucan and grain weight. To decipher the molecular mechanisms associated with malting and drought stress, Hong et al. [58] employed both transcriptomics and untargeted metabolomics to analyze barley seeds subject to drought stress and found 651 metabolites including abscisic acid, jasmonic acid, and auxin were hyper-accumulated in drought-tolerant barley genotypes. Furthermore, transcriptome analysis identified several key genes including HSP, β-glucosidase, and RLK-LRR that increased their expression in response to drought stress. Cao et al. [59] performed an integrated metabolomics and transcriptome analysis to study the drought-tolerance mechanisms in soybeans, with a special focus on the effects of melatonin. Untargeted metabolomics with transcriptomics, was able to detect 706 metabolites and 752 differentially expressed genes’ (DEGs) exposure under drought stress. The results indicated the increased biosynthesis of a number of secondary metabolites including β sitosterol, several flavonoids, and phenylpropanoids after melatonin application. Integrated metabolomics and transcriptomics analysis were carried out and identified 118 phenolic compounds and several highly expressed genes under drought stress in barley. The results identified transcriptional factor bHLH131 and fiver genes regulating the phenolic pathways under drought stress [60].

Salinity is another major abiotic stress that negatively influences crop growth and crop yield. Xu and co-workers [61] explored the salinity tolerance mechanisms of two different oat genotypes with different levels of salt tolerance. Using GC-MS/MS metabolomic methods, they were able to detect 201 differentially expressed metabolites including organic acids, amino acids, and saccharides. Transcriptomic studies indicated an upregulation in 34,030 genes involved in glycolysis and sugar and starch metabolism. Likewise, integrated metabolomics and transcriptomics analysis revealed that carbohydrate metabolism may not be required for salinity tolerance, while genes and metabolites associated with amino acid and fatty acid metabolism are key for salt tolerance in canola. The result also identified some unique metabolites produced in higher concentrations under salinity such as L-tryptophan, L-proline, alpha linolenic acid, L-phenylalanine, and L-glutamate [62].

In another study conducted by Pan and colleagues [63], the salt tolerance properties of two foxtail millet varieties were investigated. Transcriptomic data using RNASeq libraries revealed 3149 salt-responsive genes that were upregulated, and many of these were hypothesized to play a crucial role in secondary metabolism: phytohormone metabolism, redox homeostasis, and ion transport in salt-tolerant millet varieties. Untargeted metabolomics analysis detected 720 metabolites associated with pathways involved in the biosynthesis of lysophospholipid, lignin, flavonoid, and phenylpropanoid. Comparative transcriptomics and metabolic analysis were performed to study the salinity impacts on buckwheat. For this transcriptome, analysis revealed 94,950 unigenes, from which 3292 unigenes were downregulated and 4098 were upregulated under salt stress. In addition, some genes appeared to control the metabolism of nucleotide, lipid, and amino acid, and several genes were significantly involved in secondary metabolites biosynthesis including flavonoids subjected to salt stress [64]. Integrated transcriptome and metabolome analysis showed differentially expressed genes controlling the various metabolic pathways in sugar beets under salinity stress. Several genes were found to be involved in amino acid biosynthesis and carbon metabolism while metabolic profiling showed the sucrose metabolism in salt stress. Higher accumulation of allantoin controlled the gene encoding allantoinase and xanthine dehydrogenase, which were down and upregulated respectively [65].

A number of other examples of metabolomics studies have also been published exploring metabolic and transcriptomic changes arising from heat tolerance [66], cold tolerance [67], and combined stress tolerance in different crop plants [68,69].

As highlighted here, there have been a large number of studies using integrated metabolomics to understand responses to biotic/abiotic stress and to help with metabolomics-assisted selection and breeding for crop improvement. The integration of metabolomics with other omics approaches will, no doubt, significantly enhance the efficiency and accuracy of future breeding programs, particularly for the screening of novel metabolic traits associated with biotic and abiotic stress resistance.

## 5. Metabolic Engineering and Metabolic Editing

Genetic engineering of plants for improved yields, stability, and herbicide resistance dates from the early 1990s. Some of the first genetically modified (GM) crops include the FLAVR SAVR tomatoes, introduced in 1992 [70], and Roundup Ready (glyphosate resistant) soybeans, introduced in 1995 [71]. Since then, dozens of other genetically engineered crops have appeared on the market. These genetic modifications typically involved the introduction of foreign or non-plant (trans) genes into the host plant genome. As a result, the genetically engineered plants are typically called transgenic plants or genetically modified organisms (GMOs). The introduction of non-plant genes into plant genomes is largely considered “unnatural” or even unethical, and this has led to controversies and concerns about the use of transgenic GMOs, especially in Europe and other parts of the world. Indeed, strong laws and sanctions have been put in place to enforce and regulate the production and distribution of GMOs around the globe [72]. In Europe, field trails of transgenic crops are still banned for commercial purposes and the sale of GMO crops is prohibited in many markets [73]. While transgenic approaches to plant genetic engineering have contributed to some groundbreaking achievements for crop improvement, they have nevertheless incited serious public health concerns. Today, the commercialization of GMOs is subject to strict legislative laws and regulatory affairs. Typically, GMOs need 8–10 years of intensive breeding cycles and laborious regulatory assessment protocols to receive approval [73].

In contrast to transgenic plant engineering, plant metabolic engineering or metabolic editing is a promising strategy that does not require the introduction of foreign or non-plant genes into the host plant genome. Instead, metabolic engineering or metabolic editing is a method that accelerates the selective breeding process by modifying existing genes in the host plant’s specific metabolic pathways. These changes simply direct the biochemical reactions to produce more of the desired products [74]. The purpose of metabolic editing is to facilitate the biosynthesis of desirable or highly desired metabolites via the upregulation or downregulation of gene expression or by knocking out specific genes [75]. With the advent of next-generation DNA sequencing, bioinformatics tools and more advanced metabolomics tools, the discovery of putative genes governing these plant metabolic pathways has become much easier, far quicker, and more efficient. It has also allowed scientists to begin to explore how to regulate plant metabolism via metabolic editing [74]. Systematic integration of multi-omics techniques can significantly collect, annotate, analyze, and model this wealth of information. This in turn will help to identify metabolic networks and pathways that can be target for metabolic reconstruction [76].

Metabolic editing has many significant applications in crop improvement such as increased production of secondary metabolites to confer abiotic stress tolerance in crop plants [77]. Recent studies have shown that phytohormones such as cytokinin, gibberellins, ethylene, auxins, strigolactones, jasmonates, and bressionsteroids will be vital targets for metabolic editing to develop climate-resilient crops [77]. Similarly, metabolic engineering has been applied to improve resistance against invading insects, pests, fungi, and other biotic stressors in order to improve crop yield [61,78,79]. In addition, metabolic engineering has been employed to improve the flavor, quality, taste, fragrance, and enhance production of some important antioxidant and vitamins in a number of fruits and vegetables [80]. Metabolic engineering has great potential to improve the nutritive value of food products and biofuel production from plants by upregulating the desired metabolites present in different metabolic pathways.

The advent of gene editing has led to the view that metabolically edited plants can and should be considered as non-GMO [81]. This is because plants can be genome-edited with gene expression cassettes that are comprised entirely of DNA obtained from same crop species [82]. These genome-edited plants are fundamentally different from GMOs and can be commercialized without the requirement for strict regulations, in just 3–5 years. Genome-edited or metabolically edited crops may therefore be more acceptable to consumers and major regulatory stakeholders [83].

**Figure 3:**
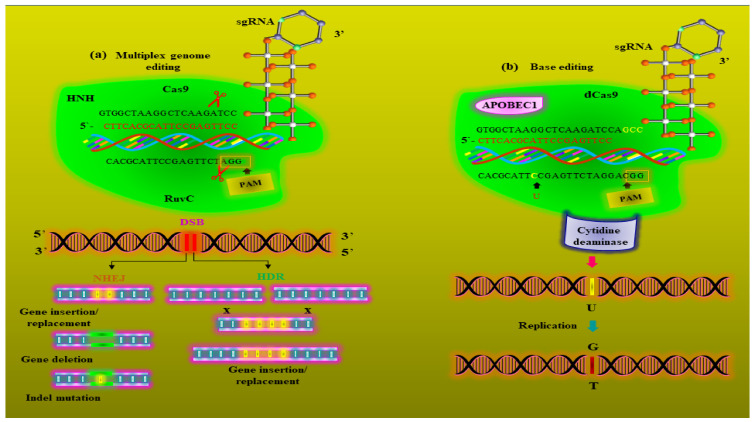
Schematic representation of how metabolic engineering/editing is done in plants through multiplexed genome editing and multiplexed base editing systems. (**a**) The CRISPR/Cas9-mediated multiplexed genome editing system consists of multiple single-guide RNAs (gRNAs), and the Cas9 protein is activated via trans-activating CRISPR RNA (tracrRNA) and guided by CRISPR RNA (crRNA) to generate site-specific double-standard breaks (DSBs) at different points on the DNA. The gRNAs detect a unique sequence of 20 nucleotides (red) and the Cas9/gRNAs complex cuts the DNA at a protospacer adjacent motif (PAM) site that is three bases upstream of the target sequence via the RuvC and HNH domains. The DSBs can be repaired either through a homology-directed repair pathway (HDR) or nonhomologous end-joining (NHEJ). (**b**) shows the modern base-editing system which can be used to edit multiple bases in different pathways for precise metabolic editing. It comprises dead Cas9 (dCas9), which is connected with cytidine deaminase (light blue). The dCas9 is guided by gRNA to target desire single base (yellow) in the DNA sequence and substitute it with another base (brown) distal to the PAM.

Generally, more than one gene is involved in a metabolic pathway, and therefore, editing only one gene at a time would not be sufficient for a useful metabolic editing result. Multi-gene or multiplexed gene editing via CRISPR/Cas9 appears to be the most promising metabolic editing system, as it allows multiple genes to be removed or inserted as shown in Figure 3. Indeed, as has been shown by a number of plant scientists, CRISPR/Cas9 can be applied to simultaneously knockout or knock-in multiple genes for almost any desired metabolic pathway [84,85]. For example, CRISPR/Cas9 mediated multiplexed genome editing has already been applied to the opium poppy in an effort to reduce the plant’s endogenous production of morphine/opium. The gene *4′OMT2*, which regulates benzylisoquinoline alkaloid (BIA) metabolism, was efficiently disrupted by CRISPR/Cas9 cassette mutagenesis to reduce the production of thebaine and morphine [86].

Metabolic editing has not only been applied to alter the production of alkaloids, but it has also been used to change the levels of other secondary metabolites, such as lycopene. Lycopene is considered as a crucial product of the carotenoid biosynthesis pathway and is known to improve the quality and color of ripe tomato fruit. In a study described by Li et al. [87], five tomato genes including *LCY-E, LCY-B1, LCY-B2, Blc,* and *SGR1* were selected for metabolic editing in the carotenoid biosynthesis pathway via multiplexed CRISPR/Cas9. The resulting metabolically edited tomato plant demonstrated a five-fold increase in the accumulation of lycopene. Similarly, in a study described by Li et al. [84], the pYLCRISPR/Cas9 toolkit was used to target five tomato genes: *SSADH, CAT9, TP1, TP2,* and *TP3* which are involved in the GABA shunt metabolic pathway. This multiplexed metabolic editing led to a 19-fold enhancement in the biosynthesis of GABA in the engineered tomato plants. GABA is produced in large amounts during fruit development in tomatoes and is considered a health-promoting functional compound. It is also involved in various regulatory pathways, and GABA homeostasis directly influences the growth and development of many plants [84].

Leguminous plants, such as soybeans, have a diverse variety of isoflavonoid metabolites, which are known to have a positive effect on human health. They also play a significant role in regulating plant-environment or plant-pathogen interactions. A recent study by Zhang et al. [88] showed how multiplexed CRISPR/Cas9-based gene editing could be used to knock out three soybean genes: *GmFNSII-1, GmF3H1,* and *GmF3H2* involved in isoflavonoid synthesis. Metabolic profiling of the metabolically edited plants revealed an enhanced production isoflavone content. Additionally, the higher levels of these isoflavones appeared to reduce the integrity of the protein coat of the soybean mosaic virus, which led to an increased resistance of the engineered soybean plant to the soybean mosaic virus.

Metabolic processes in plants can be regulated at several points including transcriptional and post-transcriptional (exon splicing) check points, as well as via translational, post-translational modifications, or protein-protein interactions. To date, most plant-based gene editing or metabolic editing efforts have focused on transcriptional modifications. However, metabolic editing may soon begin to focus on translational changes or protein engineering edits (such as amino acid changes to reduce or enhance protein stability or activity) and post-translational modifications to change protein–protein interactions [89]. The use of both protein engineering and knock-down or knock-in approaches to metabolic editing should greatly expand the capabilities of the metabolic editing toolkit. Plastid or chloroplast genome editing is another potential route which could be harnessed for plant metabolic editing. Due to its small size, homogeneity, high copy number, and high transgene expression ability, plastid genomes appear to be ideal targets for metabolic editing. Multiple genes can easily be up or downregulated and expressed or deleted in any metabolic pathway using plastid genome editing [90].

## 6. Metabolomics for Risk Assessment of Gene-Edited Crops

As noted earlier, GM or transgenic crops are currently subject to complex regulatory rules and severe market controls due to the lack of social acceptance and widespread public health concerns [91]. Outside of performing expensive targeted gene sequencing, the detection of GM crops or GM hybrid crops is difficult. Similarly, assessing the impact of trans-gene or other genetic modifications on the integrity, safety, and nutritional quality of crop plants or their impact on the environment is difficult to ascertain. As a result, the emerging field of GMO risk assessment has started to gain increased interest and traction. GMO risk-assessment is mainly performed to check the possible harmful impacts of GMOs on the environment and on animal and human health, due to the results of undesirable and non-targeted transformations in crop plants.

One of the best routes to assess the safety, phenotype, and nutritional value of transgenic crops is through taking a snapshot of their metabolomes, because metabolomics and proteomics-based analysis are more closely related to endpoint phenotypes as compared to genomics and transcriptomics. Indeed, all the manipulations performed at the genomic level for plant GMOs should ultimately be “readable” or detectable at their metabolic and nutritional level [92]. As a result, metabolomic-assisted risk assessment for transgenic crops can help with the regulation of GMOs. Indeed, there are now many examples of untargeted metabolomics being used to assess the safety of transgenic crops [93,94,95]. For example, Kogel et al. [94] performed metabolome profiling of transgenic barley and compared their metabolite profiles to conventional or naturally occurring barley cultivars. This was done to study the potential adverse effects caused by the genetic manipulation of the barley genome. The results showed that there were fewer differences in the metabolite composition of barley varieties due to engineered genetic modifications than due to the variation in natural barley varieties. Metabolic fingerprinting of transgenic wheat cultivars was performed using NMR and GC-MS. This study documented some noticeable variation in accumulation of several metabolites including asparagine, glutamine, γ-aminobutyric acid (GABA), and proline. The results showed that the metabolome of wheat varieties was influenced more significantly by environmental factors than genetic factors. In addition, any differences among transgenic cultivars and wild wheat varieties appear to be in the same range as the differences recorded between conventional lines developed in different localities [93].

Metabolomics tools have also been used to distinguish between GMO and non-GMO maize and to probe the possible risks due to changed metabolic concentrations arising from the transgenic modifications. In a study conducted by Bernillon et al. [96], two transgenic changes were investigated, including insect resistance due to Bacillus thuringiensis toxin and herbicide tolerance. These authors used metabolomic profiling via NMR and LC-ESI-QTOF-MS to look for metabolite compositional changes. The results demonstrated that no harmful metabolites were produced in any of the maize varieties, indicating that no detectable risk was introduced via the transgenic modifications. Untargeted LC-MS/MS metabolomics analysis was used to study the transgenic maize (overexpressing *Aspergillus niger* phyA2). The *phyA2* gene encodes phytase and can catalyze the hydrolysis of phytic acid to release inorganic phosphorus. The increased phosphorus and decreased phytic acid in the transgenic maize could eliminate the need for phosphorus supplementation when maize is used as animal feed. This metabolomic study demonstrated that the concentration of nine metabolites were altered in transgenic maize compared to conventional (non-transgenic) maize lines. The altered metabolites included tyrosine, glucosaminate, myo-inositol hexakisphosphate (IP6), raffinose, citrate, phosphate, indole-3-acetyl-aspartate, 2-hydroxyglutarate, and gamma-glutamyltyrosine. Only the concentration of IP6 was downregulated while the concentration of all other eight metabolites were found upregulated in transgenic maize. All nine of these metabolites are commonly found in all plant crops and their concentrations in maize fell well within the ranges found for other crop plants or other food products commonly consumed around the world. In other words, this study confirmed that the *PhyA* transgenic modification introduced no safety risk to these GMOs [97].

In 2013, Clarke and coworkers demonstrated a safety assessment analysis for transgenic soybeans (with engineered resistance to the herbicide Mesotrione) via a non-targeted metabolomics approach using UHPLC/MS/MS and GC/MS methods. This approach allowed the detection and quantification of allantoin, delta-tocopherol, myo-inositol-hexakisphosphate, ectoine, citrulline, asparagine, ribitol, phytate galactinol, gulano-1,4-lactone, genistin, and glycitin. Their results indicated that the metabolite composition of transgenic soybeans was not statistically different from that seen within the natural variation of non GMO soybean varieties [98]. Metabolic profiling of transgenic and non-transgenic potatoes has also been performed using LC-MS/MS and the results revealed, once again, that GMO varieties share a very similar metabolite composition to naturally occurring varieties [95,99]. Kusano et al. [100] used metabolomics to analyze the chemical diversity of GM tomato expressing miraculin which is a taste-improving protein. Risk assessment analysis showed about 86% of metabolites identified were already present in the LycoCyc database and more than 92% of metabolic variations were in acceptable range of safe use. In another study, metabolic and ionomic diversity of GM and conventional soybean varieties was analyzed for risk assessment. The result exhibited similar variations among the metabolic contents and no harmful metabolites were found in glyphosate herbicide resistant GM soybean [101].

**Figure 4:**
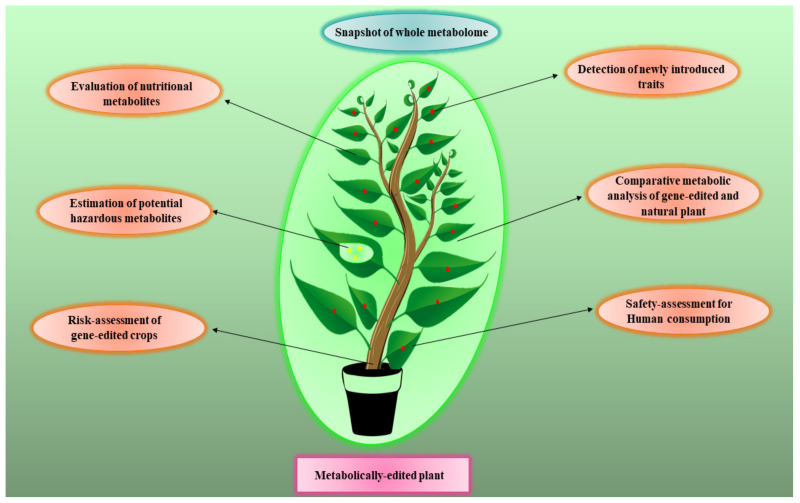
A schematic diagram portraying the benefits of metabolomics for risk-assessment of genome-edited crops.

The risk assessment of metabolically edited or gene-edited crops is likely to become a new area where metabolomics can play a role as elaborated in Figure 4. Unlike transgenic manipulations, which often lead to significant or obvious genetic changes, the consequences of gene editing or metabolic editing are more challenging (genetically) to distinguish from natural variations and normal plants. As a result, risk assessment of these more subtle genomic modifications will have to be done, once again, at the metabolic level [102]. In particular, the development of high-throughput, highly quantitative metabolomic assays will likely play a key role in assessing how metabolite editing or subtle gene editing is influencing the abundance of certain plant metabolites or other phenotypic traits compared to native strains or cultivars. Proposed applications of metabolomics in risk assessment of GM and genome edited crops will help to reduce the variety approval time and laborious protocols. It may also assist to redefine the regulatory affairs of GM/edited crop globally and assist to fast-track the crop research.

## 7. Metabolomics-Assisted Speed Breeding

Speed breeding is an emerging technology in which the generation cycle of a crop is reduced to allow the more rapid introduction of new traits into plants. This innovation was inspired by NASA, which was attempting to grow wheat plants in space and by scientists from the University of Queensland who proposed the term “speed breeding” [103]. Speed breeding works on the principle of accelerating plant growth and development using extended photoperiods under artificial lighting and tightly controlled temperature conditions. This technique mainly focuses on controlling the daily dawn and dusk photoperiod for plants in specially designed plant breeding chambers. The extended photoperiod is achieved with the help of supplementary lights by using a mixture of metal halide lights with LEDs (light emitting diodes) [104].

Typically, a 22 h light period and 2 h dark period is applied under controlled temperatures to enhance the plants’ photosynthetic activity. Under normal conditions, only 1–2 generations of a given crop can be produced per year, but with speed breeding, up to six generations of a given crops can be produced in a single year [105]. Speed breeding procedures for many crops such as wheat, canola, barley, and chickpeas have been established and are now widely available. Speed breeding is revolutionizing plant research by accelerating the breeding activities such as crossing, backcrossing, population mapping, developing transgenic pipelines, pyramiding of traits, and rapid gene identification. Moreover, speed breeding can be integrated with other technologies such as gene editing, genomic selection, high-throughput genotyping, and phenotyping to quicken the breeding programs [106].

**Figure 5:**
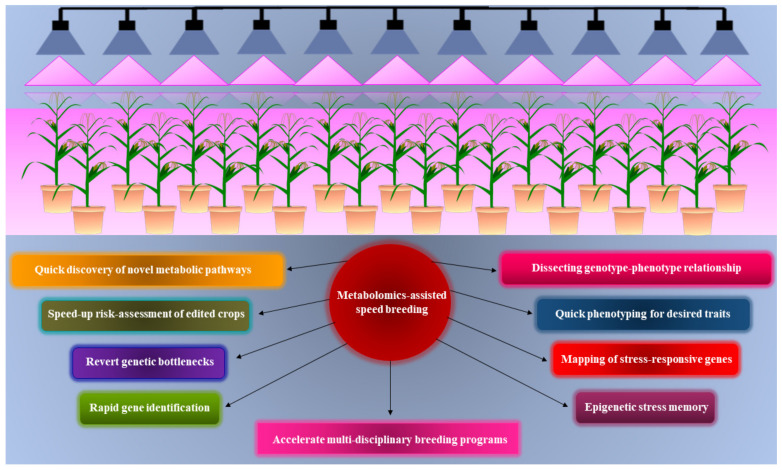
Depiction of the potential applications of metabolomics-assisted speed breeding to accelerate the crop breeding program.

Speed breeding can potentially be integrated with metabolomics approaches to fast-track crop improvement as illustrated in Figure 5. “Metabolomics-assisted speed breeding” may be applied to rapidly elucidate novel metabolic pathways or novel metabolic markers for different plant stressors. Under speed breeding conditions, a large number of accessions of any crop tested to screen the elite germplasm by identifying the resistant biomarkers. Speed breeding assisted with metabolomics expedite the process and these metabolic biomarkers can be used as a diagnostic tool to select the stress tolerant crop varieties in less time as compared to conventional breeding. In addition, this can also help the breeder to cross the elite with the elite and introducing the novel traits into the progeny with the help biomarkers in quick time.

As yet, there is no published example of metabolomics-assisted speed breeding, however we believe it is only a matter of time before examples start appearing in the literature. We expect that metabolomics-assisted speed breeding will help in the rapid phenotyping of plants, in accelerating the detection of new metabolic pathways, in rapid gene identification, and the mapping of new genes controlling different stress-responsive mechanisms in crop plants.

Metabolomics-assisted speed breeding will also provide an excellent platform to learn about the stress memory mechanism in plants and the effects of epigenetic modifications in crop plants at a metabolite level. We believe that metabolomics-assisted speed breeding will open many new avenues for crop improvement.

## 8. Conclusions and Future Directions

Climate change, reduced land quality, shrinking arable land area, and population growth are all major threats to global food security. While many of these issues require social or political solutions, there are a number of scientific options that may help address some of these challenges. As we have attempted to show in this review, a convergence of innovations in genomics, bioinformatics, and metabolomics have created a powerful version of metabolomics. Metabolomics combines advances in metabolomics technologies, genomics technologies, and bioinformatics methods to allow a much more detailed exploration of gene–metabolite–phenotype relationships. As we have shown, metabolomics has emerged as an excellent tool for crop improvement and plant biology research. In particular, metabolomics is allowing the deciphering of novel metabolic pathways, the detection and functional annotation of new metabolic genes or gene networks, the deconvolution of different stress-responsive metabolic pathways, the detection of novel genes responsible for stress tolerance, and the elucidation of the relationship between genes, metabolites, and phenotypes in a wide variety of crop plants. With advances in gene editing and metabolite editing, along with advances in speed breeding and improved GMO risk assessment, the discoveries made by metabolomics could soon lead to healthier, safer, more nutritious, more resilient, faster growing crops. Metabolomics will also play a crucial role in the characterization of gene-edited and speed-bred crops. This integration of metabolomics into the regulatory process will also prove to be very beneficial for GMO risk-assessment and commercialization of gene edited-crops. The metabolite-based safety-assessment of gene-edited crops will improve the current methods of risk-assessment of biotech crops and address public concerns related to their social acceptance.

## Figures and Tables

**Table 1:** Summary of some key studies highlighting the significance of metabolomic-assisted breeding for crop improvement.

Crop		Analytical Tool	Detected Metabolites	No. of Candidate Genes	No. of QTLs	Trait Study	Reference
Rice	mGWAS	LC-MS/MS	l-alanine, l-tyramine, threonine, leucine and histidine Syringenone, Chlorogenic acid	36	356	Nutritional value	[26]
Peral Millet		FIE-HRMS,	vitamins, antioxidants, dietary starch	738	987	Nutritional improvement	[27]
Rice		LC-MS/MS	Amino acids, flavonoids	58	24	Grain color, size, and weight	[28]
Wheat		GC-MS	L-tyrosine, pentose alcohol III, L-arginine, ornithine, oxalic acid,	25	38	Association between metabolic phenotypes	[29]
Maize		LC-MS/MS	Flavonoid, benzoxazinoid	-	-	Pathogen resistance	[30]
Maize		LC-MS	Terpenoids, benzoxazinoids, lipids, amino acids, flavonoids,	10	-	Salt tolerance	[31]
Soybean		LC-MS	Alanine, arginine, asparagine, aspartic acid, daidzein	284	144	Seed oil-related traits	[32]
Foxtail Millet		LC-ESI-MS/MS	lipids, hydroxycinnamoyl derivatives, phenolamides and flavonoids	5	237	Environment adaptation	[33]
Wheat		LC-MS/MS	Flavonoids	26	42	Flavonoid pathways	[34]
Tomato		ESI-QqTOF-MS/MS	Amino acid, alkaloids, vitamins, polyamine, polyphenol	535	-	Fruit traits	[35]
Wheat		LC-MS/MS	Betaine, deoxyinosine-5′-monophosphate	24	1005	Grains per spike, plant height	[36]
Barley		LC-MS	Glycosides, acylated glycosides of flavones, phenylpropenoic acid	-	138	Drought tolerance	[37]
Barley		MS (IC-MS/MS)	succinate, glutathione, γ-tocopherol	-	13	Drought and heat stress	[38]
Tomato		GC/MS, UPLC	Acyl-sugars, glycoalkaloids, flavonols	-	212	Fruit metabolism	[39]
Tomato		UPLC	Glycoalkaloids, acyl-sugar, hydroxycinnamates	4	679	Environmental stress tolerance	[40]
Strawberry		LC-ESI-MS	Phenolics, flavonoids, anthocyanins	-	178	Fruit quality	[41]
Rice		LC-MS/MS	L-asparagine, feruloylserotonin	35	4681	Agronomic traits	[42]

## References

[1] FAO State of Food Security and Nutrition in the World. https://www.fao.org/publications/sofi/2021/en/.

[2] Raza A., Razzaq A., Mehmood S.S., Zou X., Zhang X., Lv Y., Xu J. (2019). Impact of climate change on crops adaptation and strategies to tackle its outcome: A review. Plants.

[3] Ray D.K., Mueller N.D., West P.C., Foley J.A. (2013). Yield trends are insufficient to double global crop production by 2050. PLoS ONE.

[4] Razzaq A., Kaur P., Akhter N., Wani S.H., Saleem F. (2021). Next-generation breeding strategies for climate-ready crops. Front. Plant Sci..

[5] Aggarwal P., Vyas S., Thornton P., Campbell B.M., Kropff M. (2019). Importance of considering technology growth in impact assessments of climate change on agriculture. Glob. Food Secur..

[6] Wishart D.S., Tzur D., Knox C., Eisner R., Guo A.C., Young N., Cheng D., Jewell K., Arndt D., Sawhney S. (2007). HMDB: The human metabolome database. Nucleic Acids Res..

[7] Foito A., Stewart D. (2018). Metabolomics: A high-throughput screen for biochemical and bioactivity diversity in plants and crops. Curr. Pharm. Des..

[8] Piasecka A., Kachlicki P., Stobiecki M. (2019). Analytical methods for detection of plant metabolomes changes in response to biotic and abiotic stresses. Int. J. Mol. Sci..

[9] Razzaq A., Sadia B., Raza A., Khalid Hameed M., Saleem F. (2019). Metabolomics: A way forward for crop improvement. Metabolites.

[10] Sulpice R. (2020). Closing the yield gap: Can metabolomics be of help?. J. Exp. Bot..

[11] Wani S.H. (2019). Recent Approaches in Omics for Plant Resilience to Climate Change.

[12] Fernie A.R., Stitt M. (2012). On the discordance of metabolomics with proteomics and transcriptomics: Coping with increasing complexity in logic, chemistry, and network interactions scientific correspondence. Plant Physiol..

[13] Scossa F., Alseekh S., Fernie A.R. (2021). Integrating multi-omics data for crop improvement. J. Plant Physiol..

[14] Moreno-Risueno M.A., Busch W., Benfey P.N. (2010). Omics meet networks—using systems approaches to infer regulatory networks in plants. Curr. Opin. Plant Biol..

[15] Ryan D., Robards K. (2006). Metabolomics: The greatest omics of them all?. Anal. Chem..

[16] Villate A., San Nicolas M., Gallastegi M., Aulas P.-A., Olivares M., Usobiaga A., Etxebarria N., Aizpurua-Olaizola O. (2021). Metabolomics as a prediction tool for plants performance under environmental stress. Plant Sci..

[17] Fernie A.R., Schauer N. (2009). Metabolomics-assisted breeding: A viable option for crop improvement?. Trends Genet..

[18] Hameed M.K., Umar W., Razzaq A., Aziz T., Maqsood M.A., Wei S., Niu Q., Huang D., Chang L. (2022). Differential Metabolic Responses of Lettuce Grown in Soil, Substrate and Hydroponic Cultivation Systems under NH4^+^/NO3^−^ Application. Metabolites.

[19] Obata T., Fernie A.R. (2012). The use of metabolomics to dissect plant responses to abiotic stresses. Cell. Mol. Life Sci..

[20] Kang Z., Babar M.A., Khan N., Guo J., Khan J., Islam S., Shrestha S., Shahi D. (2019). Comparative metabolomic profiling in the roots and leaves in contrasting genotypes reveals complex mechanisms involved in post-anthesis drought tolerance in wheat. PLoS ONE.

[21] Rosato A., Tenori L., Cascante M., Carulla P.R.D.A., Dos Santos V.A.M., Saccenti E. (2018). From correlation to causation: Analysis of metabolomics data using systems biology approaches. Metabolomics.

[22] Weckwerth W. (2010). Metabolomics: An integral technique in systems biology. Bioanalysis.

[23] Weckwerth W. (2019). Toward a unification of system-theoretical principles in biology and ecology—the stochastic lyapunov matrix equation and its inverse application. Front. Appl. Math. Stat..

[24] Feng L., Wang C., Yang X., Jiao Q., Yin Y. (2022). Transcriptomics and metabolomics analyses identified key genes associated with sugar and acid metabolism in sweet and sour pomegranate cultivars during the developmental period. Plant Physiol. Biochem..

[25] Fang C., Luo J. (2019). Metabolic GWAS-based dissection of genetic bases underlying the diversity of plant metabolism. Plant J..

[26] Chen W., Gao Y., Xie W., Gong L., Lu K., Wang W., Li Y., Liu X., Zhang H., Dong H. (2014). Genome-wide association analyses provide genetic and biochemical insights into natural variation in rice metabolism. Nat. Genet..

[27] Yadav C.B., Srivastava R.K., Gangashetty P.I., Yadav R., Mur L.A., Yadav R.S. (2021). Metabolite Diversity and Metabolic Genome-Wide Marker Association Studies (mGWAS) for Health Benefiting Nutritional Traits in Pearl Millet Grains. Cells.

[28] Chen W., Wang W., Peng M., Gong L., Gao Y., Wan J., Wang S., Shi L., Zhou B., Li Z. (2016). Comparative and parallel genome-wide association studies for metabolic and agronomic traits in cereals. Nat. Commun..

[29] Matros A., Liu G., Hartmann A., Jiang Y., Zhao Y., Wang H., Ebmeyer E., Korzun V., Schachschneider R., Kazman E. (2017). Genome–metabolite associations revealed low heritability, high genetic complexity, and causal relations for leaf metabolites in winter wheat (*Triticum aestivum*). J. Exp. Bot..

[30] Zhou S., Kremling K.A., Bandillo N., Richter A., Zhang Y.K., Ahern K.R., Artyukhin A.B., Hui J.X., Younkin G.C., Schroeder F.C. (2019). Metabolome-scale genome-wide association studies reveal chemical diversity and genetic control of maize specialized metabolites. Plant Cell.

[31] Liang X., Liu S., Wang T., Li F., Cheng J., Lai J., Qin F., Li Z., Wang X., Jiang C. (2021). Metabolomics-driven gene mining and genetic improvement of tolerance to salt-induced osmotic stress in maize. New Phytol..

[32] Liu J.Y., Li P., Zhang Y.W., Zuo J.F., Li G., Han X., Dunwell J.M., Zhang Y.M. (2020). Three-dimensional genetic networks among seed oil-related traits, metabolites and genes reveal the genetic foundations of oil synthesis in soybean. Plant J..

[33] Wei W., Li S., Wang Y., Wang B., Fan G., Zeng Q., Zhao F., Xu C., Zhang X., Tang T. (2021). Metabolome-Based Genome-Wide Association Study Provides Genetic Insights Into the Natural Variation of Foxtail Millet. Front. Plant Sci..

[34] Chen J., Hu X., Shi T., Yin H., Sun D., Hao Y., Xia X., Luo J., Fernie A.R., He Z. (2020). Metabolite-based genome-wide association study enables dissection of the flavonoid decoration pathway of wheat kernels. Plant Biotechnol. J..

[35] Zhu G., Wang S., Huang Z., Zhang S., Liao Q., Zhang C., Lin T., Qin M., Peng M., Yang C. (2018). Rewiring of the fruit metabolome in tomato breeding. Cell.

[36] Shi T., Zhu A., Jia J., Hu X., Chen J., Liu W., Ren X., Sun D., Fernie A.R., Cui F. (2020). Metabolomics analysis and metabolite-agronomic trait associations using kernels of wheat (*Triticum aestivum*) recombinant inbred lines. Plant J..

[37] Piasecka A., Sawikowska A., Kuczyńska A., Ogrodowicz P., Mikołajczak K., Krystkowiak K., Gudyś K., Guzy-Wróbelska J., Krajewski P., Kachlicki P. (2017). Drought-related secondary metabolites of barley (*Hordeum vulgare* L.) leaves and their metabolomic quantitative trait loci. Plant J..

[38] Templer S.E., Ammon A., Pscheidt D., Ciobotea O., Schuy C., McCollum C., Sonnewald U., Hanemann A., Förster J., Ordon F. (2017). Metabolite profiling of barley flag leaves under drought and combined heat and drought stress reveals metabolic QTLs for metabolites associated with antioxidant defense. J. Exp. Bot..

[39] Alseekh S., Tong H., Scossa F., Brotman Y., Vigroux F., Tohge T., Ofner I., Zamir D., Nikoloski Z., Fernie A.R. (2017). Canalization of tomato fruit metabolism. Plant Cell.

[40] Alseekh S., Tohge T., Wendenberg R., Scossa F., Omranian N., Li J., Kleessen S., Giavalisco P., Pleban T., Mueller-Roeber B. (2015). Identification and mode of inheritance of quantitative trait loci for secondary metabolite abundance in tomato. Plant Cell.

[41] Labadie M., Vallin G., Petit A., Ring L., Hoffmann T., Gaston A., Potier A., Schwab W., Rothan C., Denoyes B. (2020). Metabolite quantitative trait loci for flavonoids provide new insights into the genetic architecture of strawberry (*Fragaria× ananassa*) fruit quality. J. Agric. Food Chem..

[42] Li K., Wang D., Gong L., Lyu Y., Guo H., Chen W., Jin C., Liu X., Fang C., Luo J. (2019). Comparative analysis of metabolome of rice seeds at three developmental stages using a recombinant inbred line population. Plant J..

[43] Fernandez O., Urrutia M., Bernillon S., Giauffret C., Tardieu F., Le Gouis J., Langlade N., Charcosset A., Moing A., Gibon Y. (2016). Fortune telling: Metabolic markers of plant performance. Metabolomics.

[44] Suharti W.S., Nose A., Zheng S.-H. (2016). Metabolomic study of two rice lines infected by *Rhizoctonia solani* in negative ion mode by CE/TOF-MS. J. Plant Physiol..

[45] Cuperlovic-Culf M., Wang L., Forseille L., Boyle K., Merkley N., Burton I., Fobert P.R. (2016). Metabolic biomarker panels of response to fusarium head blight infection in different wheat varieties. PLoS ONE.

[46] Dhokane D., Karre S., Kushalappa A.C., McCartney C. (2016). Integrated metabolo-transcriptomics reveals *Fusarium* head blight candidate resistance genes in wheat QTL-Fhb2. PLoS ONE.

[47] Shelp B.J., Bozzo G.G., Trobacher C.P., Zarei A., Deyman K.L., Brikis C.J. (2012). Hypothesis/review: Contribution of putrescine to 4-aminobutyrate (GABA) production in response to abiotic stress. Plant Sci..

[48] Su P., Zhao L., Li W., Zhao J., Yan J., Ma X., Li A., Wang H., Kong L. (2020). Integrated metabolo-transcriptomics and functional characterization reveals that the wheat auxin receptor TIR1 negatively regulates defense against *Fusarium graminearum*. J. Integr. Plant Biol..

[49] Kim S.B., Van den Broeck L., Karre S., Choi H., Christensen S.A., Wang G.F., Jo Y., Cho W.K., Balint-Kurti P. (2021). Analysis of the transcriptomic, metabolomic, and gene regulatory responses to *Puccinia sorghi* in maize. Mol. Plant Pathol..

[50] Karre S., Kumar A., Dhokane D., Kushalappa A.C. (2017). Metabolo-transcriptome profiling of barley reveals induction of chitin elicitor receptor kinase gene (HvCERK1) conferring resistance against *Fusarium graminearum*. Plant Mol. Biol..

[51] Pandey V., Singh M., Pandey D., Kumar A. (2018). Integrated proteomics, genomics, metabolomics approaches reveal oxalic acid as pathogenicity factor in Tilletia indica inciting Karnal bunt disease of wheat. Sci. Rep..

[52] Chen L., Wu Q., He W., He T., Wu Q., Miao Y. (2019). Combined de novo transcriptome and metabolome analysis of common bean response to Fusarium oxysporum f. sp. phaseoli infection. Int. J. Mol. Sci..

[53] Yogendra K.N., Kushalappa A.C. (2016). Integrated transcriptomics and metabolomics reveal induction of hierarchies of resistance genes in potato against late blight. Funct. Plant Biol..

[54] Guo R., Shi L., Jiao Y., Li M., Zhong X., Gu F., Liu Q., Xia X., Li H. (2018). Metabolic responses to drought stress in the tissues of drought-tolerant and drought-sensitive wheat genotype seedlings. AoB Plants.

[55] Razzaq A., Guul W., Khan M.S., Saleem F. (2021). Metabolomics: A powerful tool to study the complexity of wheat metabolome. Protein Pept. Lett..

[56] Guo X., Xin Z., Yang T., Ma X., Zhang Y., Wang Z., Ren Y., Lin T. (2020). Metabolomics Response for Drought Stress Tolerance in Chinese Wheat Genotypes (*Triticum aestivum*). Plants.

[57] Buffagni V., Vurro F., Janni M., Gullì M., Keller A.A., Marmiroli N. (2020). Shaping durum wheat for the future: Gene expression analyses and metabolites profiling support the contribution of BCAT genes to drought stress response. Front. Plant Sci..

[58] Hong Y., Ni S.-J., Zhang G.-P. (2020). Transcriptome and metabolome analysis reveals regulatory networks and key genes controlling barley malting quality in responses to drought stress. Plant Physiol. Biochem..

[59] Cao L., Jin X., Zhang Y., Zhang M., Wang Y. (2020). Transcriptomic and metabolomic profiling of melatonin treated soybean (*Glycine max* L.) under drought stress during grain filling period through regulation of secondary metabolite biosynthesis pathways. PLoS ONE.

[60] Han Z., Ahsan M., Adil M.F., Chen X., Nazir M.M., Shamsi I.H., Zeng F., Zhang G. (2020). Identification of the gene network modules highly associated with the synthesis of phenolics compounds in barley by transcriptome and metabolome analysis. Food Chem..

[61] Xu Z., Chen X., Lu X., Zhao B., Yang Y., Liu J. (2021). Integrative analysis of transcriptome and metabolome reveal mechanism of tolerance to salt stress in oat (*Avena sativa* L.). Plant Physiol. Biochem..

[62] Wang W., Pang J., Zhang F., Sun L., Yang L., Zhao Y., Yang Y., Wang Y., Siddique K.H. (2021). Integrated transcriptomics and metabolomics analysis to characterize alkali stress responses in canola (*Brassica napus* L.). Plant Physiol. Biochem..

[63] Pan J., Li Z., Dai S., Ding H., Wang Q., Li X., Ding G., Wang P., Guan Y., Liu W. (2020). Integrative analyses of transcriptomics and metabolomics upon seed germination of foxtail millet in response to salinity. Sci. Rep..

[64] Ma W., Kim J.K., Jia C., Yin F., Kim H.J., Akram W., Hu X., Li X. (2019). Comparative transcriptome and metabolic profiling analysis of buckwheat (*Fagopyrum tataricum* (L.) Gaertn.) under salinity stress. Metabolites.

[65] Liu L., Wang B., Liu D., Zou C., Wu P., Wang Z., Wang Y., Li C. (2020). Transcriptomic and metabolomic analyses reveal mechanisms of adaptation to salinity in which carbon and nitrogen metabolism is altered in sugar beet roots. BMC Plant Biol..

[66] Liu B., Kong L., Zhang Y., Liao Y. (2021). Gene and Metabolite Integration Analysis through Transcriptome and Metabolome Brings New Insight into Heat Stress Tolerance in Potato (*Solanum tuberosum* L.). Plants.

[67] Zhao Y., Zhou M., Xu K., Li J., Li S., Zhang S., Yang X. (2019). Integrated transcriptomics and metabolomics analyses provide insights into cold stress response in wheat. Crop J..

[68] Gupta A., Patil M., Qamar A., Senthil-Kumar M. (2020). ath-miR164c influences plant responses to the combined stress of drought and bacterial infection by regulating proline metabolism. Environ. Exp. Bot..

[69] Muthuramalingam P., Jeyasri R., Selvaraj A., Pandian S.K., Ramesh M. (2020). Integrated transcriptomic and metabolomic analyses of glutamine metabolism genes unveil key players in *Oryza sativa* (L.) to ameliorate the unique and combined abiotic stress tolerance. Int. J. Biol. Macromol..

[70] Redenbaugh K., Hiatt W., Martineau B., Emlay D. (1994). Regulatory assessment of the FLAVR SAVR tomato. Trends Food Sci. Technol..

[71] Padgette S.R., Kolacz K.H., Delannay X., Re D., LaVallee B., Tinius C., Rhodes W., Otero Y., Barry G., Eichholtz D. (1995). Development, identification, and characterization of a glyphosate-tolerant soybean line. Crop Sci..

[72] Razzaq A., Saleem F., Kanwal M., Mustafa G., Yousaf S., Imran Arshad H.M., Hameed M.K., Khan M.S., Joyia F.A. (2019). Modern trends in plant genome editing: An inclusive review of the CRISPR/Cas9 toolbox. Int. J. Mol. Sci..

[73] Waltz E. (2016). CRISPR-edited crops free to enter market, skip regulation. Nat. Biotechnol..

[74] Tatsis E.C., O’Connor S.E. (2016). New developments in engineering plant metabolic pathways. Curr. Opin. Biotechnol..

[75] Korkina L. (2007). Phenylpropanoids as naturally occurring antioxidants: From plant defense to human health. Cell Mol. Biol..

[76] Jamil I.N., Remali J., Azizan K.A., Nor Muhammad N.A., Arita M., Goh H.-H., Aizat W.M. (2020). Systematic multi-omics integration (MOI) approach in plant systems biology. Front. Plant Sci..

[77] Ganjewala D., Kaur G., Srivastava N. (2019). Metabolic engineering of stress protectant secondary metabolites to confer abiotic stress tolerance in plants. Molecular Approaches in Plant Biology and Environmental Challenges.

[78] Kumar V., Baweja M., Singh P.K., Shukla P. (2016). Recent developments in systems biology and metabolic engineering of plant–microbe interactions. Front. Plant Sci..

[79] Yang T., Stoopen G., Yalpani N., Vervoort J., de Vos R., Voster A., Verstappen F.W., Bouwmeester H.J., Jongsma M.A. (2011). Metabolic engineering of geranic acid in maize to achieve fungal resistance is compromised by novel glycosylation patterns. Metab. Eng..

[80] Blancquaert D., De Steur H., Gellynck X., Van Der Straeten D. (2017). Metabolic engineering of micronutrients in crop plants. Ann. N. Y. Acad. Sci..

[81] Woo J.W., Kim J., Kwon S.I., Corvalán C., Cho S.W., Kim H., Kim S.-G., Kim S.-T., Choe S., Kim J.-S. (2015). DNA-free genome editing in plants with preassembled CRISPR-Cas9 ribonucleoproteins. Nat. Biotechnol..

[82] Lu Y., Zhu J.-K. (2017). Precise editing of a target base in the rice genome using a modified CRISPR/Cas9 system. Mol. Plant.

[83] Schouten H.J., Krens F.A., Jacobsen E. (2006). Cisgenic plants are similar to traditionally bred plants: International regulations for genetically modified organisms should be altered to exempt cisgenesis. EMBO Rep..

[84] Li R., Li R., Li X., Fu D., Zhu B., Tian H., Luo Y., Zhu H. (2018). Multiplexed CRISPR/Cas9-mediated metabolic engineering of γ-aminobutyric acid levels in Solanum lycopersicum. Plant Biotechnol. J..

[85] Najera V.A., Twyman R.M., Christou P., Zhu C. (2019). Applications of multiplex genome editing in higher plants. Curr. Opin. Biotechnol..

[86] Alagoz Y., Gurkok T., Zhang B., Unver T. (2016). Manipulating the biosynthesis of bioactive compound alkaloids for next-generation metabolic engineering in opium poppy using CRISPR-Cas 9 genome editing technology. Sci. Rep..

[87] Li X., Wang Y., Chen S., Tian H., Fu D., Zhu B., Luo Y., Zhu H. (2018). Lycopene is enriched in tomato fruit by CRISPR/Cas9-mediated multiplex genome editing. Front. Plant Sci..

[88] Zhang P., Du H., Wang J., Pu Y., Yang C., Yan R., Yang H., Cheng H., Yu D. (2020). Multiplex CRISPR/Cas9-mediated metabolic engineering increases soya bean isoflavone content and resistance to soya bean mosaic virus. Plant Biotechnol. J..

[89] Swinnen G., Goossens A., Colinas M. (2019). Metabolic editing: Small measures, great impact. Curr. Opin. Biotechnol..

[90] Schindel H.S., Piatek A.A., Stewart C.N., Lenaghan S.C. (2018). The plastid genome as a chassis for synthetic biology-enabled metabolic engineering: Players in gene expression. Plant Cell Rep..

[91] Farre G., Twyman R.M., Christou P., Capell T., Zhu C. (2015). Knowledge-driven approaches for engineering complex metabolic pathways in plants. Curr. Opin. Biotechnol..

[92] Christ B., Pluskal T., Aubry S., Weng J.-K. (2018). Contribution of untargeted metabolomics for future assessment of biotech crops. Trends Plant Sci..

[93] Baker J.M., Hawkins N.D., Ward J.L., Lovegrove A., Napier J.A., Shewry P.R., Beale M.H. (2006). A metabolomic study of substantial equivalence of field-grown genetically modified wheat. Plant Biotechnol. J..

[94] Kogel K.-H., Voll L.M., Schäfer P., Jansen C., Wu Y., Langen G., Imani J., Hofmann J., Schmiedl A., Sonnewald S. (2010). Transcriptome and metabolome profiling of field-grown transgenic barley lack induced differences but show cultivar-specific variances. Proc. Natl. Acad. Sci. USA.

[95] Shepherd L.V.T., Hackett C.A., Alexander C.J., McNicol J.W., Sungurtas J.A., Stewart D., McCue K.F., Belknap W.R., Davies H.V. (2015). Modifying glycoalkaloid content in transgenic potato–Metabolome impacts. Food Chem..

[96] Bernillon S., Maucourt M., Deborde C., Chéreau S., Jacob D., Priymenko N., Laporte B., Coumoul X., Salles B., Rogowsky P.M. (2018). Characterization of GMO or glyphosate effects on the composition of maize grain and maize-based diet for rat feeding. Metabolomics.

[97] Rao J., Yang L., Guo J., Quan S., Chen G., Zhao X., Zhang D., Shi J. (2016). Metabolic changes in transgenic maize mature seeds over-expressing the *Aspergillus niger* phyA2. Plant Cell Rep..

[98] Clarke J.D., Alexander D.C., Ward D.P., Ryals J.A., Mitchell M.W., Wulff J.E., Guo L. (2013). Assessment of genetically modified soybean in relation to natural variation in the soybean seed metabolome. Sci. Rep..

[99] Catchpole G.S., Beckmann M., Enot D.P., Mondhe M., Zywicki B., Taylor J., Hardy N., Smith A., King R.D., Kell D.B. (2005). Hierarchical metabolomics demonstrates substantial compositional similarity between genetically modified and conventional potato crops. Proc. Natl. Acad. Sci. USA.

[100] Kusano M., Redestig H., Hirai T., Oikawa A., Matsuda F., Fukushima A., Arita M., Watanabe S., Yano M., Hiwasa-Tanase K. (2011). Covering chemical diversity of genetically-modified tomatoes using metabolomics for objective substantial equivalence assessment. PLoS ONE.

[101] Kusano M., Baxter I., Fukushima A., Oikawa A., Okazaki Y., Nakabayashi R., Bouvrette D.J., Achard F., Jakubowski A.R., Ballam J.M. (2015). Assessing metabolomic and chemical diversity of a soybean lineage representing 35 years of breeding. Metabolomics.

[102] Fraser P.D., Aharoni A., Hall R.D., Huang S., Giovannoni J.J., Sonnewald U., Fernie A.R. (2020). Metabolomics should be deployed in the identification and characterization of gene-edited crops. Plant J..

[103] Watson A., Ghosh S., Williams M.J., Cuddy W.S., Simmonds J., Rey M.-D., Hatta M.A.M., Hinchliffe A., Steed A., Reynolds D. (2018). Speed breeding is a powerful tool to accelerate crop research and breeding. Nat. Plants.

[104] Ghosh S., Watson A., Gonzalez-Navarro O.E., Ramirez-Gonzalez R.H., Yanes L., Mendoza-Suárez M., Simmonds J., Wells R., Rayner T., Green P. (2018). Speed breeding in growth chambers and glasshouses for crop breeding and model plant research. Nat. Protoc..

[105] Jähne F., Hahn V., Würschum T., Leiser W.L. (2020). Speed breeding short-day crops by LED-controlled light schemes. Theor. Appl. Genet..

[106] Hickey L.T., Hafeez A.N., Robinson H., Jackson S.A., Leal-Bertioli S.C., Tester M., Gao C., Godwin I.D., Hayes B.J., Wulff B.B. (2019). Breeding crops to feed 10 billion. Nat. Biotechnol..

